# Sediment descriptions and geochemical analyses of radiocarbon-dated deposits from the vicinity of Göbekli Tepe—A dataset

**DOI:** 10.1016/j.dib.2020.106012

**Published:** 2020-07-11

**Authors:** Moritz Nykamp, Daniel Knitter, Brigitta Schütt

**Affiliations:** a Freie Universität Berlin, Institute of Geographical Sciences, Malteserstr. 74-100, 12249 Berlin, Germany; b Department of Geography, Physical Geography, Christian-Albrechts-Universität zu Kiel, Ludewig-Meyn-Str. 14, 24118 Kiel, Germany

**Keywords:** Sediment sequences, Depositional architecture, Geochemical bulk parameters, Facies interpretation, Late Holocene geomorphodynamics

## Abstract

This dataset comprises the detailed descriptions and laboratory measurements of sediment profiles from the semi-arid environs of the Pre-Pottery Neolithic site Göbekli Tepe in southeastern Turkey—one of the oldest monumental structures of humankind dating to c. 11.5–10 ka BP. Focus of the descriptions are the architectural elements of the deposits allowing to conduct facies interpretations and the reconstruction of different depositional environments. This is supported by bulk geochemical sediment analyses (pH, electrical conductivity, magnetic susceptibility, and loss on ignition) and the determination of total and inorganic carbon contents and chemical element concentrations. The Late Holocene chronology is based on radiocarbon dating of charcoal pieces and bulk samples containing organic matter from buried organic-rich topsoil horizons and soil sediments. Lithic artifacts from the Pre-Pottery Neolithic provide additional age estimates. Nykamp et al. [1] provide the synthesis that is based on the presented datasets.

**Specifications Table**

**Table:** 

Subject	Earth-Surface Processes
Specific subject area	Detailed description of i) sediment sequences, ii) the depositional architecture and iii) geochemical parameters.
Type of data	Images Figures Map
How data were acquired	The presented data were collected in the field conducting detailed descriptions of sediment profiles. Samples for additional laboratory analyses were systematically collected and analyzed in the Laboratory for Physical Geography of the Freie Universität Berlin. Radiocarbon dating was done TÜBİTAK National 1 MV AMS Laboratory and the Poznań Radiocarbon Laboratory. Macroscopic sediment descriptions follow Ad-Hoc-AG Boden [2], Schoeneberger et al. [3], and Tucker [4]. Colors were recorded according to Munsell soil color charts and converted to RGB-values. This together with digitization of clasts allows providing realistic drawings of the sediment profiles. Laboratory analyses followed standard protocols. For pH and electrical conductivity determinations pH and EC/Temperature checkers (Hanna Instruments) were used. Loss on ignition was determined according to Dean [5] and Heiri et al. [6]. Mass specific magnetic susceptibility was assessed using the MS2B sensor (Bartington Instruments) according to Dearing [7]. Total carbon contents were determined with a TruSpec CHN (Leco) analyzer and total inorganic carbon contents were measured with a Carmhograph C-16 (Wösthoff) carbon analyzer as described in Müller et al. [8]. Concentrations of chemical elements were determined with a portable energy-dispersive X-ray fluorescence spectrometer (p-ED-XRF; Thermo Scientific Niton XL3t).
Data format	Raw and analyzed data are provided in the descriptions and illustrations of the studied sediment profiles.
Parameters for data collection	Field description of sediment composition, sedimentary structures and architectural elements of gravels. Determination of bulk geochemical parameters (pH, electrical conductivity, loss on ignition, and magnetic susceptibility), and total carbon, total inorganic carbon and element concentrations for fine material (< 2 mm).
Description of data collection	Macroscopic sediment descriptions and systematic sampling were done in the field. Collected samples were prepared and analyzed in the Laboratory for Physical Geography of the Freie Universität Berlin.
Data source location	Southeastern Turkey; c. 12 km northeast of the city of Şanlιurfa. GPS coordinates of profile locations are presented in Nykamp et al. [1].
Data accessibility	With the article
Related research article	M. Nykamp, D. Knitter, B. Schütt, Late Holocene geomorphodynamics in the vicinity of Göbekli Tepe, SE Turkey. Catena 195 (2020) 104759. doi: 10.1016/j.catena.2020.104759

## Value of the Data

• Detailed and systematic descriptions of sediment profiles with particular focus on their depositional architecture allow conducting facies interpretation. This, together with geochemical analyses and a geochronology facilitate reconstructions of past depositional environments.
• Readers find raw and analyzed datasets associated with the study of Nykamp et al. [1]. Considering the here presented datasets allows to examine the summary presented by Nykamp et al. [1] and to better follow their discussion.
• The dataset itself as well as the approach used to systematically collect the data can be used as a basis for future studies—not only in the environs of Göbekli Tepe—that combine facies interpretation with geochemical and chronological data.

## 1 Data Description

**Fig. 1:**
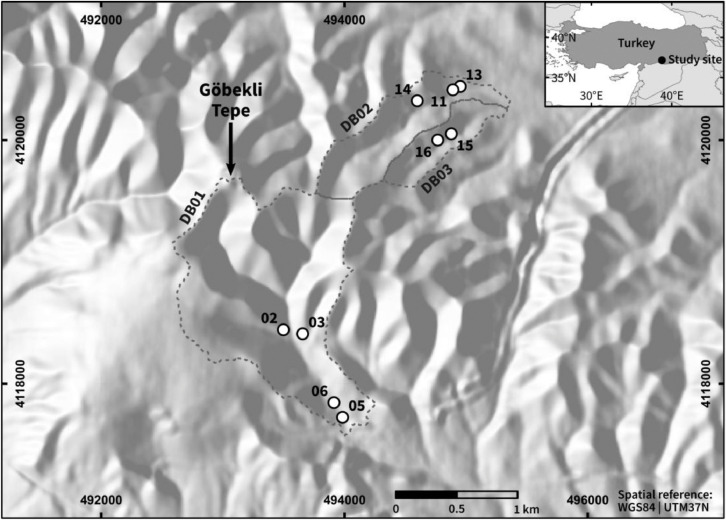
Overview map of the study site in the close vicinity of the Early Neolithic site Göbekli Tepe in southeastern Anatolia (shaded relief with threefold vertical exaggeration; elevation data based upon TanDEM-X digital elevation model with 12 m² pixel size, © DLR, 2017). The map indicates the locations of the sediment sequences within the three studied drainage basins (DB01–03). The numbers refer to the profile ID “GT” in the text.

The profiles are located in three different drainage basins of small intermittent streams that drain the limestone plateau of Göbekli Tepe and the adjacent slopes (Fig. 1). The profiles GT02–GT06 are located in drainage basin 1, south of Göbekli Tepe. The profiles GT11–GT14 and the profiles GT15 and GT16 are located in the two drainage basins 2 and 3, east of Göbekli Tepe. The profiles are presented according to their locations in the respective drainage basins in downstream direction. The two key profiles GT04 and GT12 (not shown in Fig. 1) are presented in detail in Nykamp et al. [1]. Geochemical parameters of the presented sediment profiles are included in the Supplementary material.

### 1.1 Drainage basin 1

The profiles GT02 and GT03 are located along the lower courses of two tributary valleys (first order valleys). Along the intermittent drainage ways the colluvial deposits at the foot slopes are dissected forming natural exposures. GT02 is located on the left side (downstream direction) of the western tributary valley c. 1.4 km SSE of Göbekli Tepe and GT03 is located on the right side (downstream direction) of the eastern tributary valley c. 1.5 km SSE of Göbekli Tepe. The profiles GT05 (c. 2.2 km SSE of Göbekli Tepe) and GT06 (c. 2.1 km SSE of Göbekli Tepe) are located along the thalweg of the main valley (receiving stream) in undercut slope positions.

**Fig. 2:**
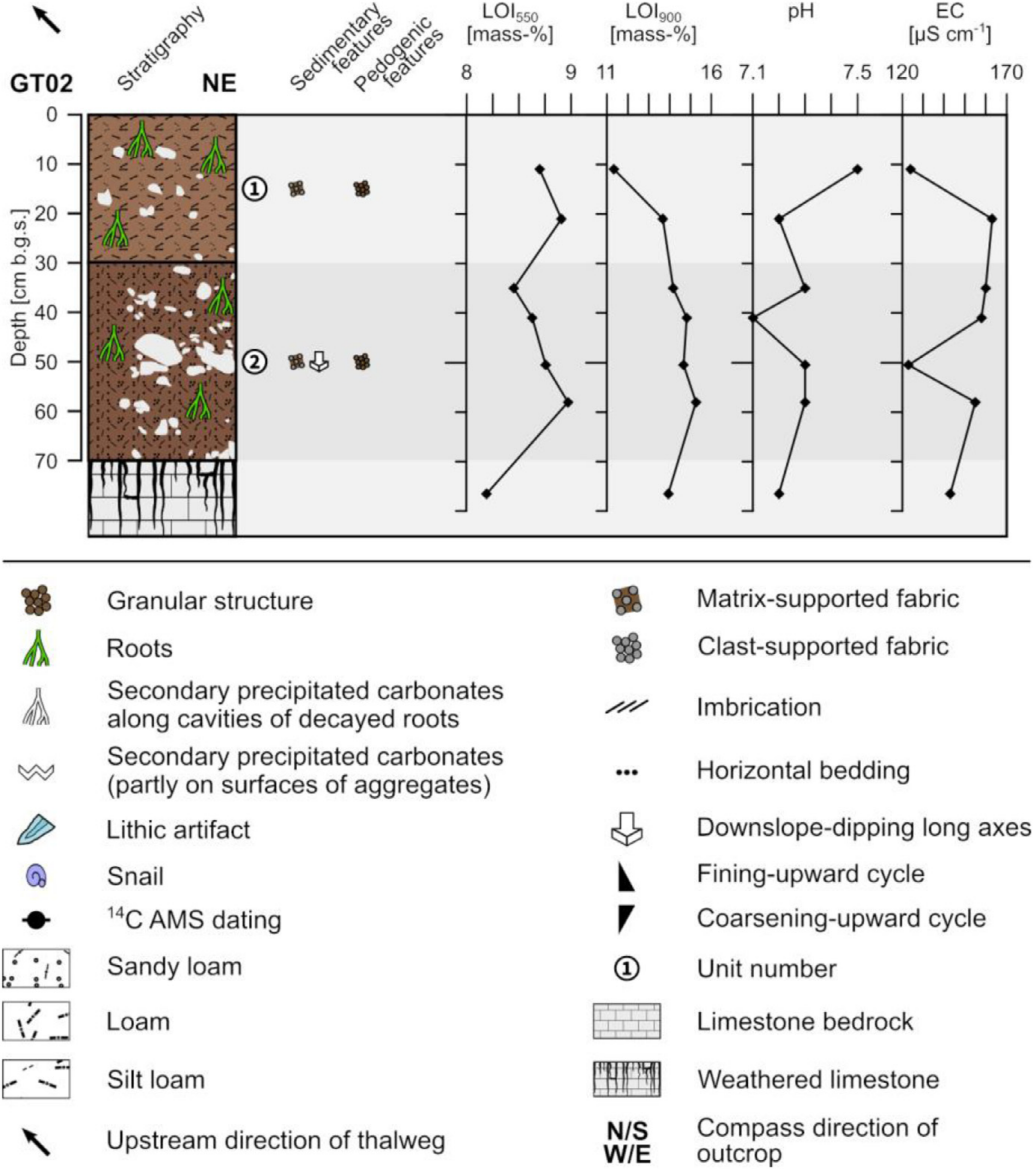
Profile drawing and bulk parameters of GT02. The legend applies to all following profile drawings.

The profile GT02 (493,518 E, 4,118,389N; UTM 37N) has a total thickness of 70 cm and shows two units. At 70 cm depth the sediments show a diffuse boundary with the underlying weathered limestone (Fig. 2).

Unit 1 (0–30 cm b.g.s.) represents the present-day plowing horizon and shows a diffuse boundary with unit 2. It is composed of slightly compacted reddish brown (5YR 5/4) silt loam with a granular structure and common roots. The occurring clasts account for c. 10% and comprise of angular to sub-rounded limestone pebbles and occasionally sub-rounded limestone cobbles forming a matrix-supported fabric. The clasts are poorly sorted and ungraded.

Unit 2 (30–70 cm b.g.s.) consists of moderately compacted reddish brown (5YR 4/4) sandy loam with a granular structure and common roots. C. 30% angular to sub-rounded limestone pebbles and some, mostly platy, limestone cobbles occur. The clasts are ungraded and poorly sorted forming a matrix-supported fabric. The platy cobbles tend to have down-slope dipping long axes.

The LOI_550_ values are rather high in unit 1, slightly drop towards the top of unit 2 and slightly increase with depth in the course of unit 2; the underlying weathered limestone shows the lowest LOI_550_ values. The LOI_900_ values increase in unit 1 from top to bottom and remain rather high throughout the profile (Fig. 2). The pH values are slightly alkaline and show in unit 1 a decrease towards the bottom, the lowest value at c. 40 cm depth and little variation for the rest of the profile. The electrical conductivity is low in the uppermost part of unit 1 and at c. 50 cm b.g.s. while higher values characterize the rest of the profile.

**Fig. 3:**
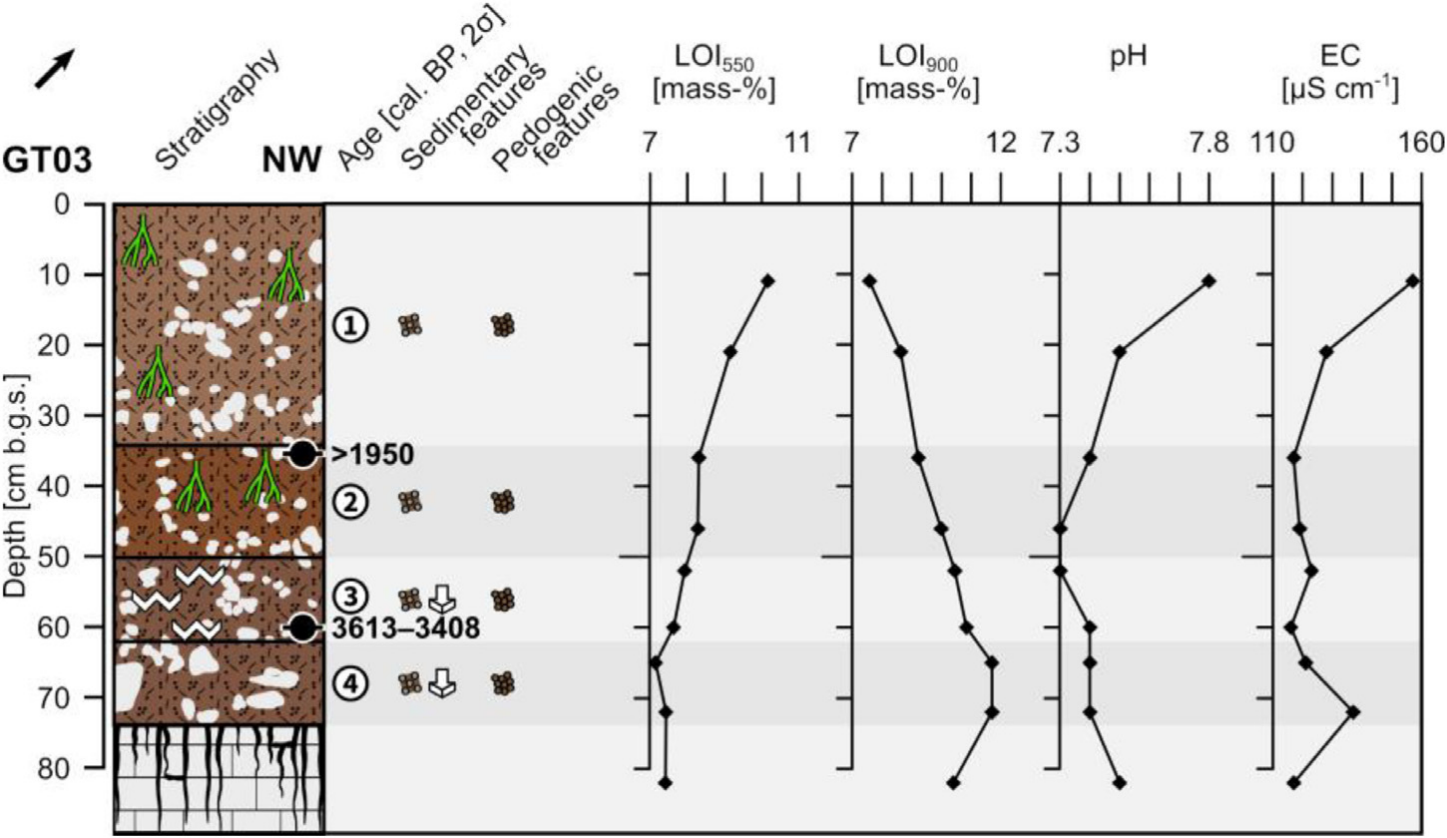
Profile drawing and bulk parameters of GT03. See Fig. 2 for legend.

The profile GT03 (493,656 E, 4,118,322N; UTM 37N) has a total thickness of 74 cm and shows four units. A diffuse boundary with the underlying weathered limestone occurs at 74 cm depth (Fig. 3).

Unit 1 (0–34 cm b.g.s.) represents the present-day plowing horizon and shows a diffuse boundary with unit 2. It is composed of very slightly compacted reddish brown (5YR 5/4) sandy loam with a granular structure and common roots. The occurring clasts account for c. 10% and comprise sub-rounded to rounded limestone pebbles forming a matrix-supported fabric. The clasts are moderately sorted and ungraded.

Unit 2 (34–50 cm b.g.s.) consists of slightly compacted yellowish red (5YR 4/6) sandy loam with a granular to coherent structure and common roots. The matrix consists of c. 10% sub-angular to sub-rounded limestone pebbles. The clasts are ungraded and moderately sorted forming a matrix-supported fabric. The boundary with unit 3 is distinct. The radiocarbon dating of a bulk sample containing organic matter at the top of unit 2 yields a modern age.

Unit 3 (50–62 cm b.g.s.) is composed of moderately compacted reddish brown (5YR 4/4) sandy loam with a granular structure and few roots. Secondary precipitated carbonates form coatings on the surfaces of the aggregates. The occurring clasts account for c. 15% and comprise sub-rounded and partly platy sub-angular limestone pebbles that are poorly sorted and ungraded. The platy clasts occasionally show down-slope dipping of their long axes. The radiocarbon dating of a bulk sample containing organic matter at the bottom of unit 3 yields an age of 3613–3408 cal. a BP (1664–1459 a BCE). Unit 3 shows a gradual boundary with unit 4.

Unit 4 (62–74 cm b.g.s.) is similar to unit 3, but the material is more compacted and the carbonate coatings are absent.

The LOI_550_ values constantly drop from top to bottom, while the LOI_900_ values steadily rise (Fig. 3). The pH values are slightly alkaline and show a decrease from the uppermost part in unit 1 to the top of unit 3 and a slight increase below. The electrical conductivity in unit 1 constantly decreases from top to bottom and remains low from the beginning unit 2 until the weathered limestone; except for a higher value at the bottom of unit 4.

**Fig. 4:**
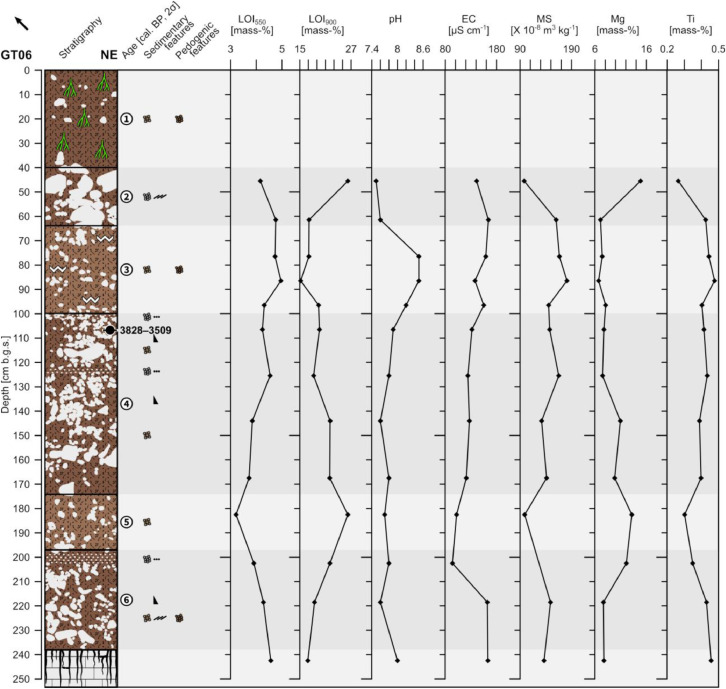
Profile drawing and bulk parameters of GT06. See Fig. 2 for legend.

The profile GT06 (493,946 E, 4,117,785N; UTM 37N) has a total thickness of 238 cm and shows six units. At 238 cm depth a gradual boundary with the underlying weathered limestone occurs that was recorded until 255 cm depth (Fig. 4).

Unit 1 (0–40 cm b.g.s.) consists of non-compacted reddish brown (5YR 4/4) sandy loam with a granular structure and common roots. Common sub-rounded to rounded fine limestone pebbles are incorporated forming a matrix-supported fabric. Unit 1 represents the present-day plowing horizon and shows a clear boundary with unit 2.

Unit 2 (40–64 cm b.g.s.) shows a clast-supported fabric that is composed of poorly sorted and ungraded sub-rounded to well-rounded pebbles and cobbles. Commonly, the clasts show imbrication. The fine material consists of reddish brown (5YR 4/4) sandy loam. Unit 2 has a sharp erosive contact with unit 3.

Unit 3 (64–100 cm b.g.s.) is composed of heavily compacted reddish brown (5YR 5/4) sandy loam with a granular structure and without roots. Secondary precipitated carbonates form weakly developed coatings on the surfaces of the aggregates. Partly platy and partly sub-rounded limestone pebbles account for c. 20% forming a matrix-supported fabric. The clasts are ungraded and moderately sorted. Unit 3 shows a clear boundary to unit 4.

Unit 4 (100–174 cm b.g.s.) is characterized by alternating layers of sub-rounded to well-rounded limestone pebbles and cobbles with little fine material that consists of reddish brown (5YR 4/4) sandy loam. The pebble-dominated layers are moderately to well sorted, horizontally bedded and have a clast-supported fabric. The cobble-dominated layers have more fine material and are poorly to moderately sorted forming a matrix-supported fabric. In combination, the two cobble- and two pebble-dominated layers are normal graded and show two fining-upward cycles. Unit 4 shows a sharp erosive contact with unit 5. The radiocarbon dating of a bulk sample containing organic matter at the top of unit 4 yields an age of 3828–3509 cal. a BP (1879–1560 a BCE).

Unit 5 (174–197 cm b.g.s.) consists of heavily compacted reddish brown (5YR 5/4) sandy loam. Sub-rounded, ungraded and poorly sorted pebbles and few cobbles are incorporated forming a matrix-supported fabric. Unit 5 shows a clear boundary with unit 6.

Unit 6 (197–238 cm b.g.s.) consists of sub-angular to rounded pebbles and cobbles that are normal graded forming one fining-upward cycle. The pebble-dominated upper part is well sorted and shows horizontal bedding. It has very little fine material and a clast-supported fabric. The cobble-dominated lower part is poorly to moderately sorted and shows more fine material, a matrix-supported fabric and occasionally imbrication. The fine material consists of reddish brown (5YR 4/4) sandy loam having a granular structure in the lower part of the unit. A single boulder-sized limestone clast occurs between 210 and 227 cm depth.

The underlying weathered limestone (238–255 cm b.g.s.) consists of a mixture of reddish gray (5YR 5/2) loam with common manganese nodules and heavily weathered limestone bedrock. The fine material is regarded to be the in-situ weathering product.

Unit 1 was not sampled. Units 2 and 3 show relatively high LOI_550_ values that decrease below 90 cm b.g.s. and reach a minimum value in unit 5. The LOI_900_ values show a pattern that is roughly opposed to the one of the LOI_550_ (Fig. 4). Throughout the profile the pH values are slightly alkaline and show, except for increased pH values in unit 3, little variation. The electrical conductivity shows rather constantly decreasing values with depth and a marked increase in unit 6 overlying the weathered limestone. The magnetic susceptibility shows minimum values at the top of unit 2 and in unit 5; beyond, magnetic susceptibility varies with a slight decrease from top to bottom. Within unit 2 the Mg concentrations drop with depth while the Ti concentrations increase. Below, until c. 140 cm b.g.s., the Mg values remain low and the Ti values rather high. Towards unit 5 the Mg values increase and the Ti values decrease and in the lower part of unit 6 the Mg values are low again, while the Ti values are rather high.

**Fig. 5:**
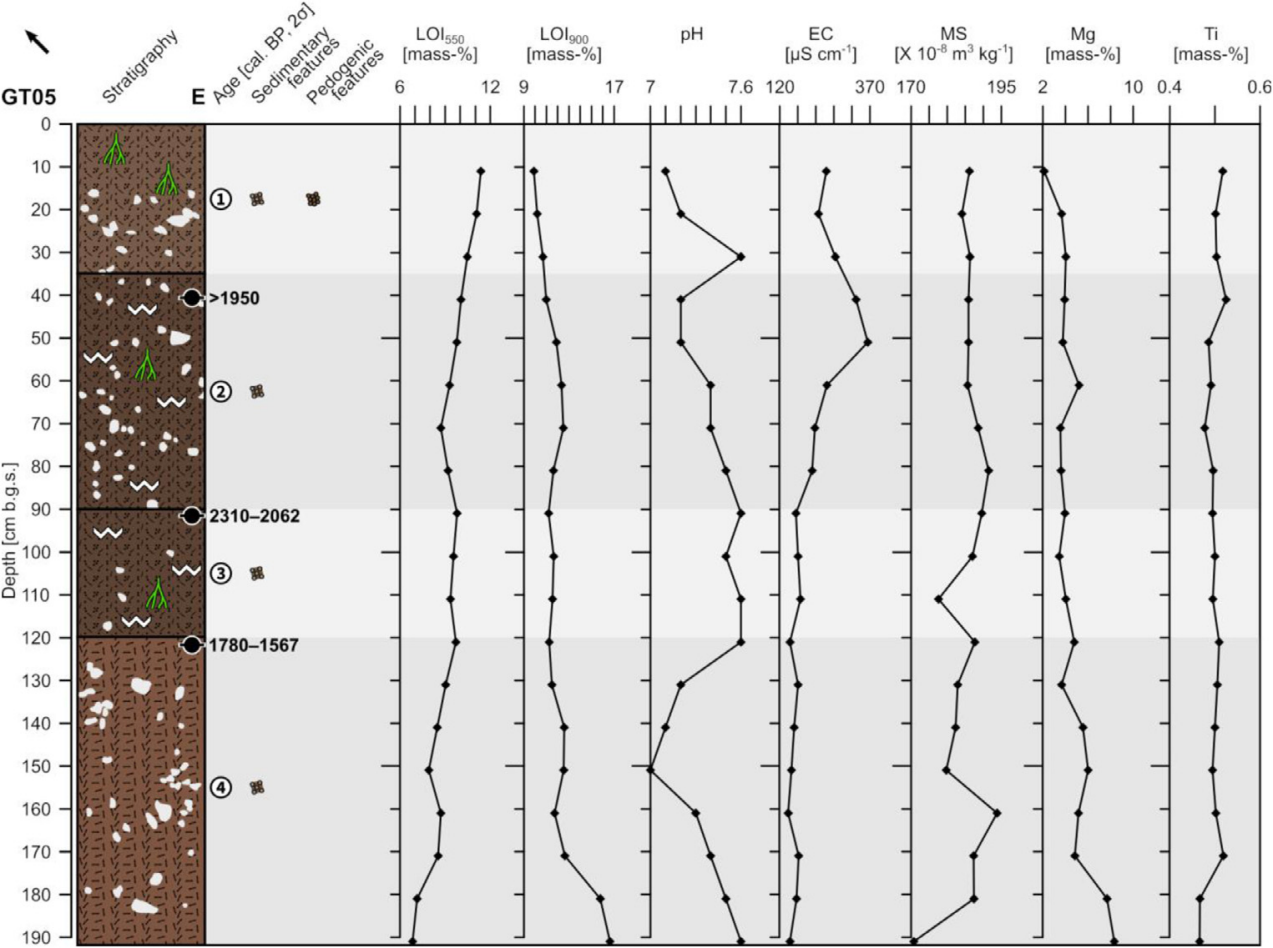
Profile drawing and bulk parameters of GT05. See Fig. 2 for legend.

The profile GT05 (494,017 E, 4,117,664N; UTM 37N) has a total thickness of 192 cm and shows four units (Fig. 5). The underlying bedrock is not reached.

Unit 1 (0–35 cm b.g.s.) represents the present-day plowing horizon and shows a diffuse boundary with unit 2. It consists of slightly compacted reddish brown (5YR 4/3) sandy loam with a granular structure and few roots. Angular to sub-rounded limestone pebbles are incorporated (c. 20%) forming a matrix-supported fabric. The clasts are moderately sorted and ungraded.

Unit 2 (35–90 cm b.g.s.) is composed of heavily compacted dark reddish brown (5YR 3/3) sandy loam with very few roots. Occasionally, secondary precipitated carbonates occur. The clasts show the same characteristics as in unit 1. The radiocarbon dating of a bulk sample containing organic matter at the top of unit 2 yields a modern age. Unit 2 shows a diffuse boundary with unit 3.

Unit 3 (90–120 cm b.g.s.) mainly shows the same characteristics as unit 2, but contains less pebbles and only locally few roots. The radiocarbon dating of a bulk sample containing organic matter at the top of unit 3 yields an age of 2310–2062 cal. a BP (361–113 a BCE). The boundary between unit 3 and unit 4 is diffuse.

Unit 4 (120–192 cm b.g.s.) consists of heavily compacted reddish brown (5YR 4/4) loam. Angular to sub-angular coarse pebbles account for c. 10% and fine pebbles are less abundant. The clasts consist of limestone, are moderately sorted and ungraded; the fabric is matrix-supported. The radiocarbon dating of a bulk sample containing organic matter at the top of unit 4 yields an age of 1780–1567 cal. a BP (171–383 a CE).

The LOI_550_ shows a rather constant decrease with depth while the same time the LOI_900_ slightly increases with minor variations, except for a strong increase at the bottom of unit 4. The pH values are slightly alkaline throughout the profile showing an increase at the bottom of unit 1 and a decrease between c. 130–160 cm b.g.s. (Fig. 5). Except of the increase in the upper half of unit 2 the electrical conductivity shows little variation. The magnetic susceptibility shows little variation above c. 100 cm b.g.s. and larger variations below. The Mg and Ti values show little variation, besides the slight increase of Mg and drop of Ti at the bottom of unit 4.

### 1.2 Drainage basin 2

Profile GT14 is located in an undercut slope position on the right side (downstream direction) of the valley (first order valley) in the lower part of drainage basin 2, c. 1.7 km ENE of Göbekli Tepe. At this foot slope location colluvial sediments are dissected forming a natural exposure. Further downstream, at the position where the valley enters the undulating plain, are the locations of the profiles GT11 and GT13. They are located close to each other in a c. 100 m long artificial pit that forms an exposure, c. 2 km ENE of Göbekli Tepe (Fig. 1).

**Fig. 6:**
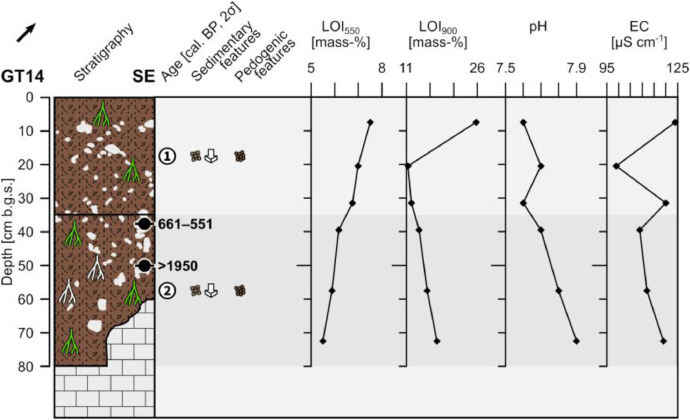
Profile drawing and bulk parameters of GT14. See Fig. 2 for legend.

The profile GT14 (494,632 E, 4,120,264N; UTM 37N) has a total thickness of 80 cm and shows two units with only few distinguishing characteristics and a diffuse boundary. On the right side of the outcrop the underlying limestone bedrock occurs below 80 cm depth and below 60 cm depth on the left side (Fig. 6).

Unit 1 (0–35 cm b.g.s.) consists of reddish brown (5YR 4/4) sandy loam that is slightly compacted in the upper 16 cm and heavily compacted below. The material shows a granular structure and is commonly interfused by roots. Up to 10% of angular to sub-rounded, poorly sorted and ungraded limestone pebbles are incorporated forming a matrix-supported fabric. Downslope-dipping of the long axes of the clasts occurs.

Unit 2 (35–80 cm b.g.s.) has the same characteristics as unit 1, but it shows more roots and c. 20% of very angular to sub-angular, partly platy limestone pebbles whose long axes occasionally dip downslope. Secondary precipitated carbonates occur along cavities of decayed roots. The radiocarbon dating of a bulk sample containing organic matter at the top of unit 2 yields an age of 661–551 cal. a BP (1290–1399 a CE) and the dating of a piece of charcoal, embedded at 50 cm depth, yields a modern age.

The LOI_550_ values show a constant decrease with depth and the LOI_900_ reaches a maximum value close to the surface, while below 20 cm b.g.s. LOI_900_ values slightly increase with depth (Fig. 6). The pH values are slightly alkaline and roughly increase constantly with depth. The electrical conductivity shows, except for the minimum value at c. 20 cm b.g.s., little variation.

**Fig. 7:**
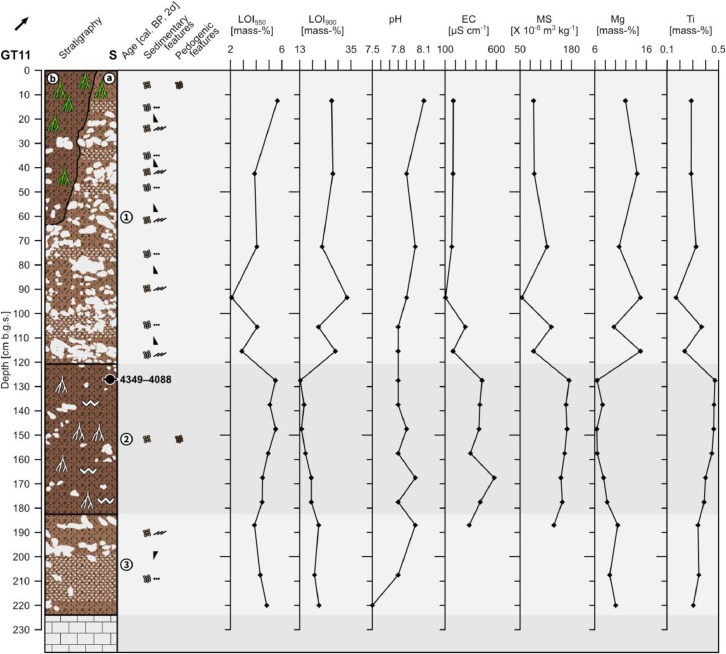
Profile drawing and bulk parameters of GT11. See Fig. 2 for legend.

**Fig. 8:**
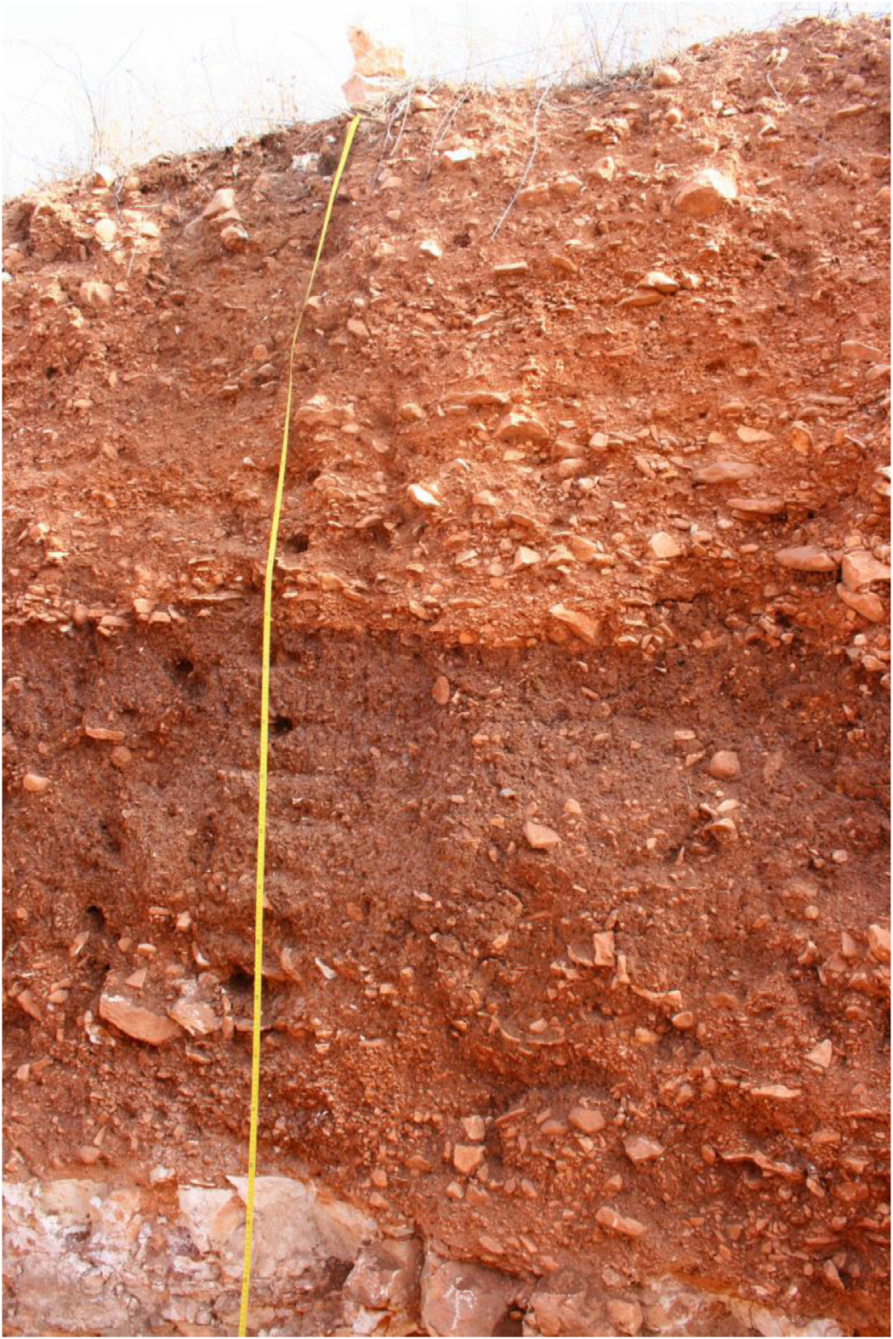
Photograph of profile GT11.

The profile GT11 (494,922 E, 4,120,378N; UTM 37N) has a total thickness of 224 cm and shows three distinguishable units. At 224 cm depth the sediments show a sharp contact with the underlying limestone bedrock (Figs. 7 and 8).

Unit 1 (0–121 cm b.g.s.) is in the upper 63 cm vertically subdivided into sections a) and b). Unit 1a) represents the most recent sediment unit that can be followed along the outcrop. In the area where GT11 was recorded a dug-out pit occurs that reaches 63 cm depth. This pit is backfilled with material that shows different characteristics than the surrounding sediments of unit 1a) and has sharp boundaries with unit 1a). The backfilled material represents unit 1b) that was sampled in the uppermost 58 cm. At 121 cm depth unit 1 shows a sharp erosive contact with unit 2.

Unit 1a) shows alternating layers of limestone pebbles and cobbles. The pebbles are usually sub-angular to rounded and partly show horizontal bedding. The pebble-dominated layers are mostly moderately to well sorted, frequently contain very little fine material and usually have a clast-supported fabric. The cobbles are angular to sub-rounded, often platy and regularly show imbrication. The cobble-dominated layers tend to be moderately to poorly sorted and often contain more fine material than the pebble layers. The fabric is partly matrix-supported and partly clast-supported. The pebble and the cobble layers form five fining-upward cycles, each normally graded. The fine material consists of reddish brown (5YR 5/4) sandy loam that shows a granular structure in the uppermost part.

Unit 1b) consists of reddish brown (5YR 4/4) sandy loam with a granular structure. The upper 25 cm show many roots, which decrease in frequency towards the bottom. The compactness of the sediments increases from top to bottom; the uppermost 25 cm are slightly compacted while the lower 33 cm are moderately to heavily compacted. The incorporated clasts are usually sub-angular to rounded limestone pebbles and very few cobbles that account for c. 30% in total. The clasts are ungraded and poorly sorted forming a matrix-supported fabric.

Unit 2 (121–182 cm b.g.s.) consists of heavily compacted reddish brown (5YR 4/4) sandy loam with a granular structure. Secondary precipitated carbonates occur along segments of root cavities and partly form coatings on the surfaces of the aggregates. The incorporated clasts consist of angular to rounded limestone pebbles and few cobbles and account for c. 10%. The clasts are poorly sorted and ungraded and the fabric is matrix-supported. The radiocarbon dating of a bulk sample containing organic matter in the upper part of unit 2 yields an age of 4349–4088 cal. a BP (2400–2139 a BCE).

Unit 3 (182–224 cm b.g.s.) is formed by up to 90% limestone pebbles and cobbles that are inversely graded forming one coarsening-upward cycle. The pebbles are sub-angular to rounded, usually show horizontal bedding and are moderately to well sorted. Only little fine material occurs in this pebble-dominated unit; the fabric is clast-supported. The cobbles towards the top of the unit are poorly sorted, angular to sub-rounded, often platy and frequently show imbrication; the fabric is matrix-supported. The fine material is composed of reddish brown (5YR 5/4) sandy loam.

The LOI_550_ decreases in unit 1 from the top to c. 120 cm b.g.s. with oscillating values in the lower part. It abruptly increases in unit 2 and remains high until the bottom of unit 3. The LOI_900_ shows high, strongly varying values in unit 1 and abruptly drops in unit 2 and again slightly increases from top to bottom in unit 3 (Fig. 7). The pH values are slightly alkaline throughout the profile and the electrical conductivity shows low values in unit 1 and substantially increased values in unit 2. The pattern of the magnetic susceptibility resembles the one of the electrical conductivity. The Mg and Ti concentrations show an inverse pattern. Mg concentrations are high, but oscillating, in unit 1, low in unit 2 and slightly increase towards the bottom in unit 3. Ti concentrations are rather low in unit 1 and oscillate in the lower part; highest values are reached in unit 2 that decrease below c. 160 cm depth.

**Fig. 9:**
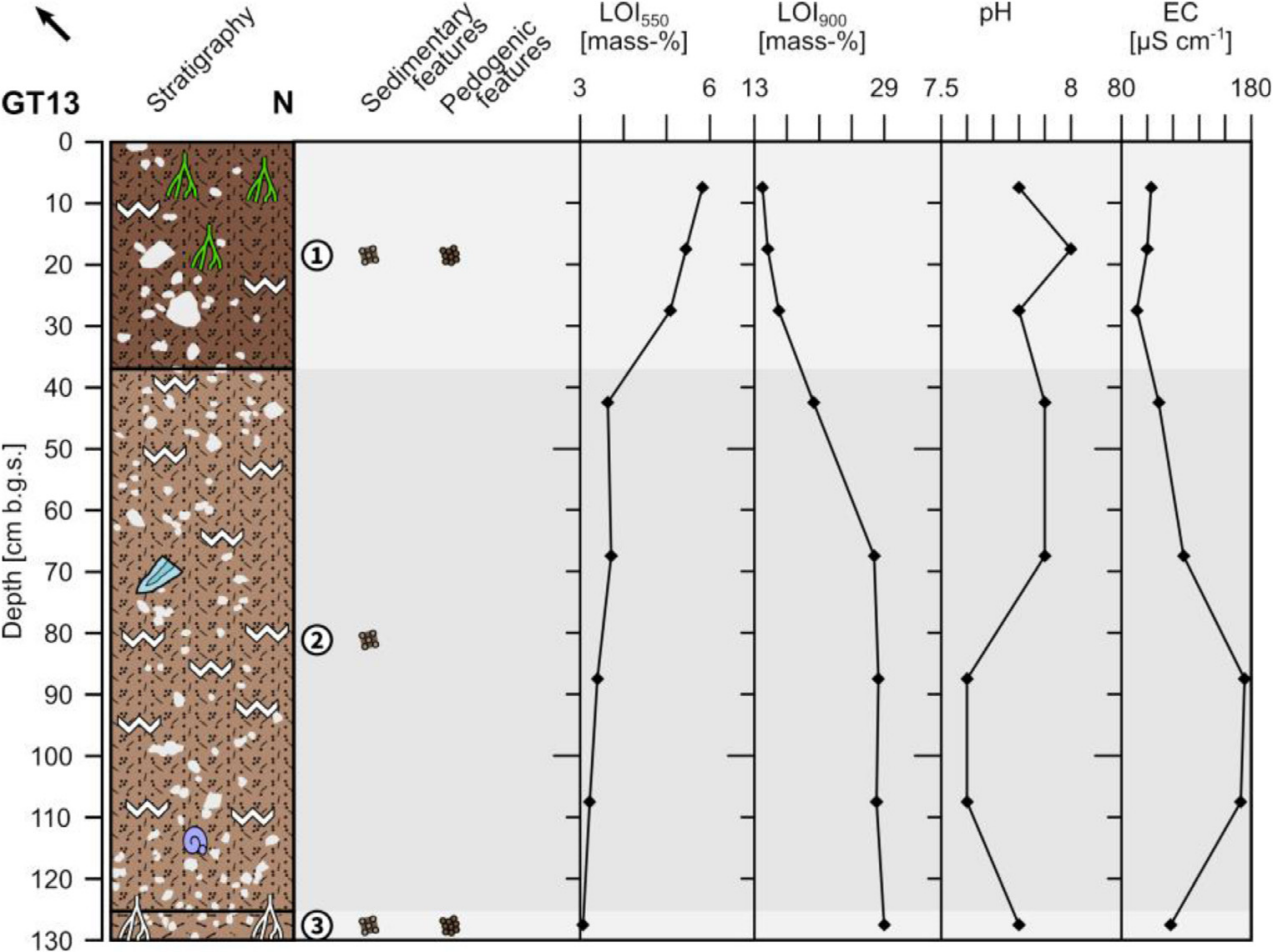
Profile drawing and bulk parameters of GT13. See Fig. 2 for legend.

**Fig. 10:**
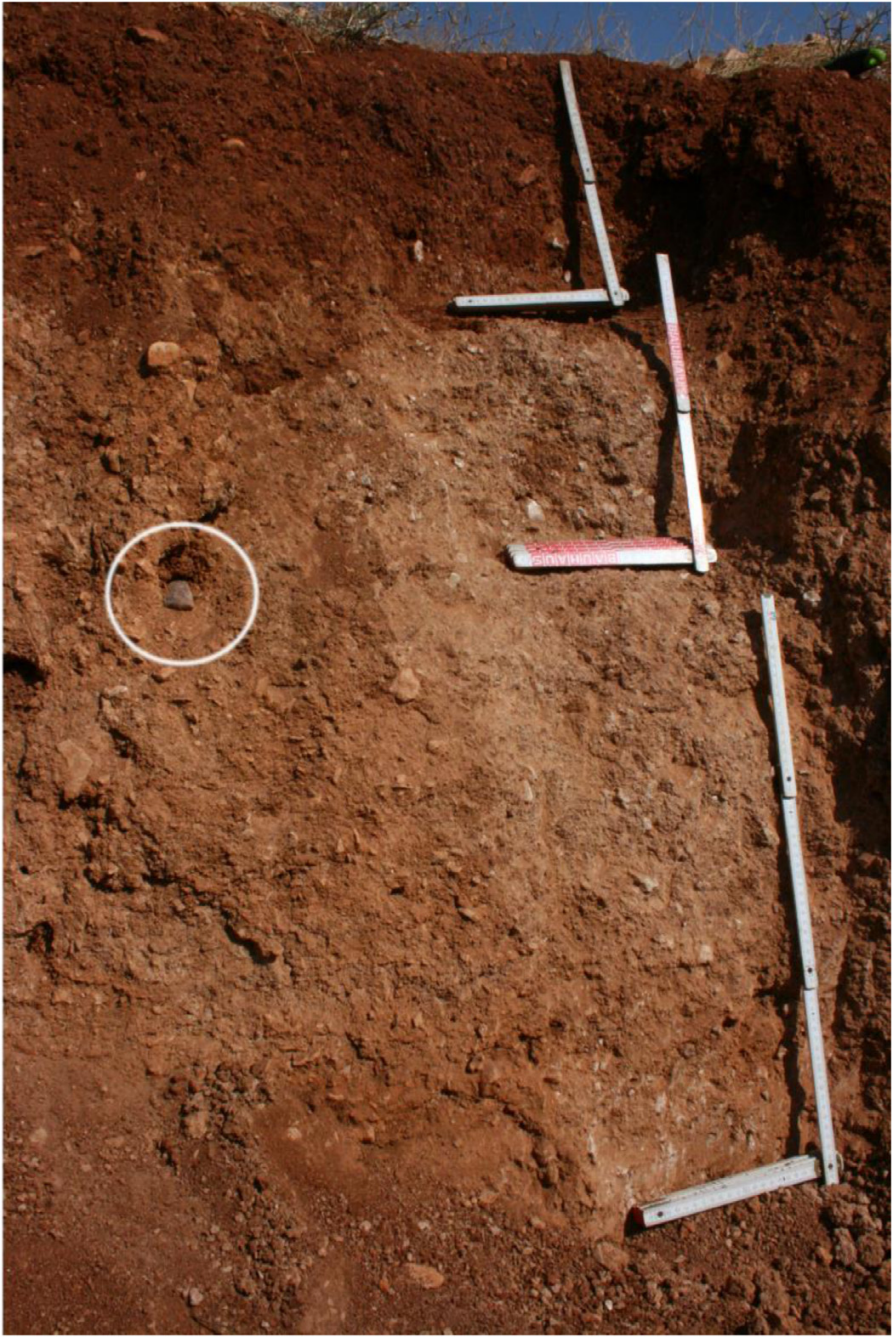
Photograph of profile GT13. Please note the highlighted lithic scraper at 70 cm depth.

The profile GT13 (494,957 E, 41,203,894N; UTM 37N) has a total thickness of 130 cm and shows three distinguishable units (Figs. 9 and 10). The underlying bedrock is not reached.

Unit 1 (0–37 cm b.g.s.) consists of reddish brown (5YR 4/4) sandy loam with a granular structure. Occasionally secondary precipitated carbonates form coatings on the surfaces of the aggregates. The uppermost 10 cm show common roots that decrease in frequency towards the bottom. The material is slightly compacted at the top and heavily compacted at the bottom of the unit. The occurring limestone pebbles (c. 10%) are poorly sorted, ungraded and angular to rounded forming a matrix-supported fabric. Below c. 30 cm depth the color of the sediment becomes increasingly gray forming a clear boundary with unit 2.

Unit 2 (37–125 cm b.g.s.) is composed of light reddish brown (5YR 6/4) sandy loam that is strongly interfused with secondary precipitated carbonates forming a heavily compacted calcrete. Up to 20% sub-angular to rounded, moderately sorted and ungraded limestone pebbles are incorporated forming a matrix-supported fabric. Roots do not occur in unit 2. A c. 4.5 cm long patinized lithic scraper from the Pre-Pottery Neolithic period was found at 70 cm depth. Unit 2 has a gradual boundary with unit 3.

Unit 3 (125–130 cm b.g.s.) consists of light reddish brown (5YR 6/4) heavily compacted silt loam that has a granular structure and does not contain roots. Secondary precipitated carbonates occur along cavities of decayed roots. Sub-rounded to rounded limestone pebbles (c. 5%) are incorporated in the fine material forming a matrix-supported fabric.

The LOI_550_ values constantly decrease in unit 1 from top to bottom and remain low in units 2 and 3 until the bottom of the profile. The LOI_900_ is low in unit 1, increases in the upper part of unit 2 and remains high in the lower part of the profile (Fig. 9). The pH values are slightly alkaline and show a slight decrease in the lower part of unit 2, below 70 cm b.g.s; the electrical conductivity roughly mirrors the course of the pH values.

### 1.3 Drainage basin 3

The profiles GT16 and GT15 are located along the thalweg of the valley (first order valley) in the drainage basin 3, east of Göbekli Tepe. Both profiles are located in undercut slope positions on the right side (downstream direction) of the drainage way that dissects the colluvial deposits at the foot slope. The profile locations are approximately half way between the headwater area and the transition towards the margin of the undulating plain and between c. 1.8 km (GT15) and c. 1.9 km (GT16) E of Göbekli Tepe (Fig. 1).

**Fig. 11:**
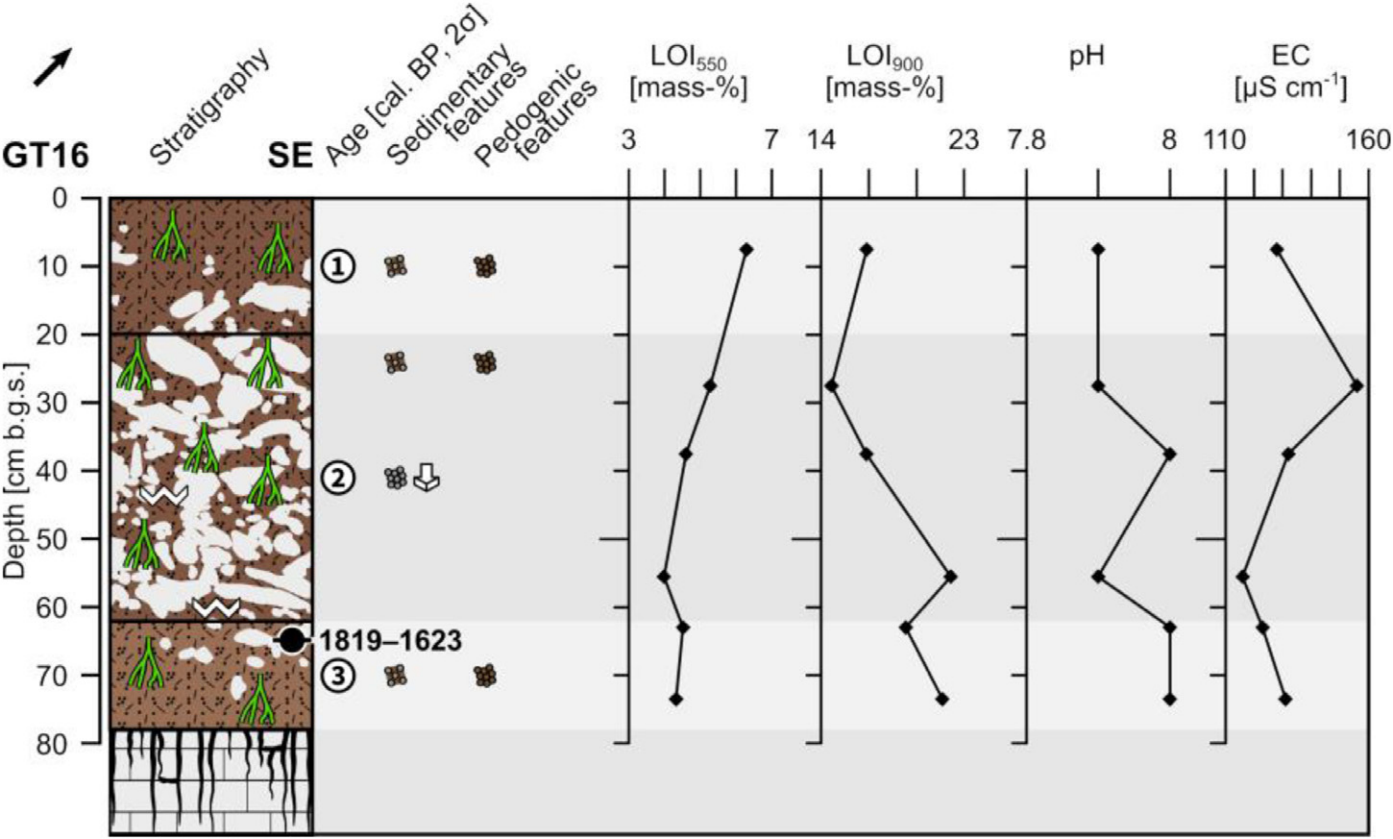
Profile drawing and bulk parameters of GT16. See Fig. 2 for legend.

**Fig. 12:**
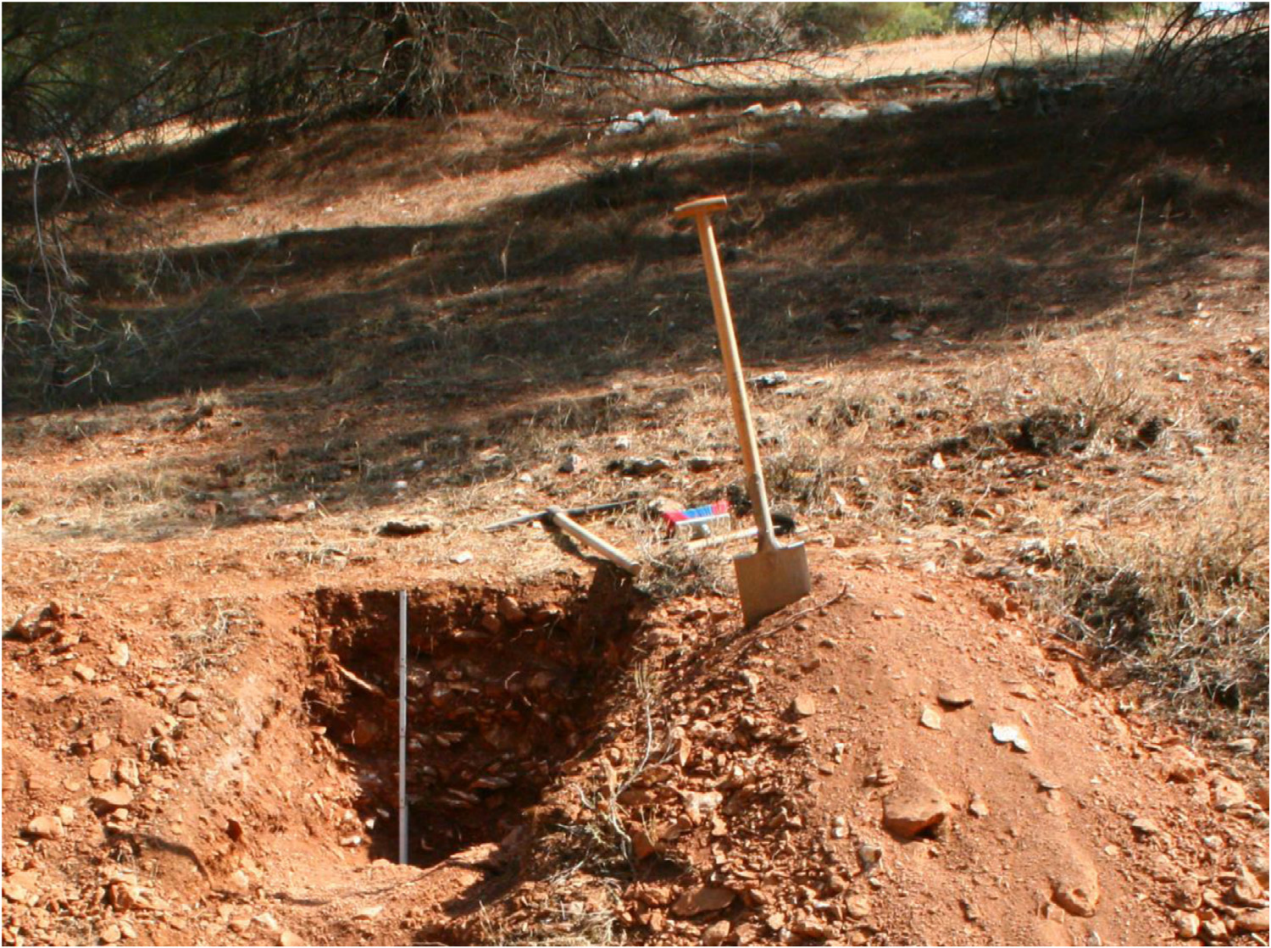
Photograph of profile GT16.

The profile GT16 (494,800 E, 4,119,940 N; UTM 37 N) has a total thickness of 78 cm and shows three units. Below 78 cm depth a clear boundary with the underlying weathered limestone occurs (Figs. 11 and 12).

Unit 1 (0–20 cm b.g.s.) is composed of slightly compacted reddish brown (5YR 4/4) sandy loam with a granular structure. Roots occur commonly. Limestone pebbles and cobbles (c. 20%) are incorporated forming a matrix-supported fabric. The clasts are ungraded and poorly sorted. The pebbles are usually sub-angular and the cobbles have mostly a sub-rounded to rounded shape. Unit 1 shows a clear boundary with unit 2.

Unit 2 (20–62 cm b.g.s.) consists of moderately compacted reddish brown (5YR 4/4) sandy loam with many roots. The fine material has a granular structure and occasionally shows coatings of secondary precipitated carbonates on the surfaces of the aggregates. Ungraded and poorly sorted limestone clasts account for c. 70%; the pebbles are sub-angular to rounded and the cobbles usually angular to sub-angular, mostly platy and often show downslope-dipping of their long axes. The fabric is partly matrix- and partly clast-supported. Unit 2 has a sharp boundary with unit 3.

Unit 3 (62–78 cm b.g.s.) consists of heavily compacted reddish brown (5YR 5/4) sandy loam with a granular structure and common roots. Angular to sub-angular, ungraded and moderately sorted limestone pebbles (c. 10%) occur forming a matrix-supported fabric. The radiocarbon dating of a bulk sample containing organic matter at the top of unit 3 yields an age of 1819–1623 cal. a BP (132–328 a CE).

The LOI_550_ values show a constant decrease with depth, while, by contrast, the LOI_900_ shows increasing values with depth. The pH values are slightly alkaline and vary slightly. The electrical conductivity shows, except for a maximum value at c. 30 cm b.g.s., little variation (Fig. 11).

**Fig. 13:**
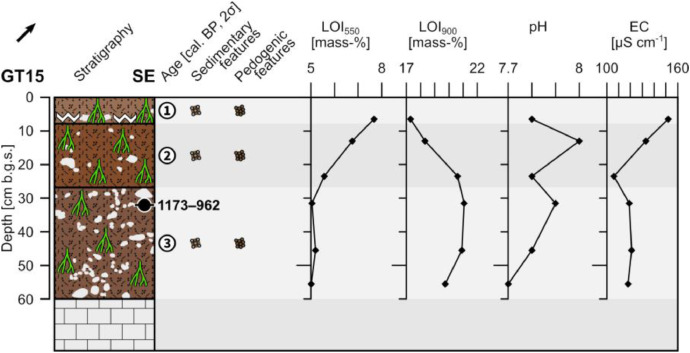
Profile drawing and bulk parameters of GT15. See Fig. 2 for legend.

The profile GT15 (494,912 E, 4,119,993 N; UTM 37 N) has a total thickness of 60 cm and shows three units. The underlying limestone bedrock occurs below 60 cm depth and shows a sharp contact with the overlying sediments (Fig. 13).

Unit 1 (0–8 cm b.g.s.) consists of slightly compacted reddish brown (5YR 5/4) sandy loam with a granular structure and common roots. Secondary precipitated carbonates occur close to the bottom. Up to 20% ungraded and well sorted fine and medium sized limestone pebbles occur; coarse pebbles occur only occasionally. The fine and medium pebbles are sub-rounded to rounded and the coarse pebbles are angular. The fabric is matrix-supported. The presence of secondary precipitated carbonates at the basis forms a clear boundary with unit 2.

Unit 2 (8–27 cm b.g.s.) has mainly the same characteristic as unit 1, but it is strongly interfused by roots and lacks secondary precipitated carbonates. The color is yellowish red (5YR 4/6), the material is moderately compacted and few cobble-sized clasts occur. Unit 2 shows a diffuse boundary with unit 3.

Unit 3 (27–60 cm b.g.s.) is composed of heavily compacted reddish brown (5YR 4/4) sandy loam that has a granular structure. The material is strongly interfused by roots and contains c. 30% angular to sub-angular limestone pebbles and cobbles that are ungraded and moderately sorted forming a matrix-supported fabric. The radiocarbon dating of a bulk sample containing organic matter in the upper part of unit 3 yields an age of 1173–962 cal. a BP (777–988 a CE).

The LOI_550_ values constantly drop from top to bottom in the uppermost 30 cm of the profile and remain low in unit 3. The LOI_900_ shows from top to bottom increasing values within the units 1 and 2 and increased values in unit 3 (Fig. 13). The pH values are slightly alkaline and show the lowest value close to the contact with bedrock. The electrical conductivity constantly decreases from top to bottom within units 1 and 2 and remain rather low in unit 3.

## 2 Experimental design, materials and methods

Electrical conductivity (µS cm^−1^) and pH values were determined in a 1:2.5 solution of 10 g of air-dried sediment and 25 ml of bi-distilled water using a handheld pH checker (Hanna Instruments) with a resolution of 0.1 for pH and an EC/Temperature checker (Hanna Instruments) for electrical conductivity.

The loss on ignition at 550 °C and at 900 °C was determined following the procedure of Dean [5]. The dried samples (105 °C, for four hours) were weighed, heated in a muffle furnace at 550 °C for four hours, cooled down in a desiccator, and weighed again. Thereafter, the same procedure was repeated at 900 °C. The loss on ignition is calculated according to Heiri et al. [6] and provides estimates of the amount of organic matter at 550 °C and of the carbonate content at 900 °C [5]. Quality control is assessed using an internal standard (STD1: LOI_550_ = 13.81 ± 0.24 mass-% C; LOI_900_ = 11.08 ± 0.25 mass-% C) and calcium carbonate (CaCO_3_: LOI_900_ = 44.04 ± 0.51 mass-% C) as reference materials with accuracy (% diff) and reproducibility (% RSD) as quality measures [9]. The reference materials provided accurate (< 5% diff) and reproducible (< 2% RSD) measurements.

Low frequency (0.46 kHz) volume specific magnetic susceptibility (κ) was determined with the MS2B sensor (Bartington Instruments) using air-dried and weighed sub-samples packed in 10 cm³ sample containers. Mass specific susceptibility (X 10^−8^ m³ kg^−1^) is calculated by dividing the volume susceptibility by the bulk density of the samples [7].

The total carbon (TC) contents were measured using the TruSpec CHN (Leco) analyzer whereby the samples are dry combusted at 950 °C in an O_2_ atmosphere and evolving CO_2_ fluxes are quantified by infrared spectroscopy. The total inorganic carbon (TIC) contents were analyzed using the Carmhograph C-16 (Wösthoff) carbon analyzer. The samples are treated with 42.5% H_3_PO_4_ acid at 80 °C and the CO_2_, evolving from the dissolved carbonates, is quantified through the change of conductivity of a 0.05 M NaOH solution. The total organic carbon (TOC) contents correspond to the difference between TIC and TC [8]. The quality is controlled using certified reference materials (Leco 502–309 = 11.89 ± 0.44 mass-% C; Leco 502–308 = 2.3 ± 0.06 mass-% C; Leco 502–062 = 0.926 ± 0.04 mass-% C) for TC measurements and internal standards (STD4.1 = 4.1 mass-% C; STD2.4 = 2.4 mass-% C) and calcium carbonate (CaCO3 = 12.01 ± 0.14 mass-% C) for TIC measurements. The reliability of results is documented by accurate (0.46% diff for TC; 2.43% diff for TIC) and reproducible (0.16% RSD for TC; 3.9% RSD for TIC) measurements.

The concentrations of chemical elements were determined using a portable energy-dispersive X-ray fluorescence spectrometer (p-ED-XRF; Thermo Scientific Niton XL3t). Powdered and oven-dried (105 °C) samples were placed in 32 mm sample cups and covered with mylar foil (0.4 µm). A certified reference material (CRM; NCS DC 73,389) was re-measured after every ten sample measurements to assess data quality. CRM measurements yielded accurate (1.22% diff) and reproducible (0.8% RSD) results for Ti, but show limitations for Mg (25.2% diff; 24% RSD).

## Declaration of Competing Interest

The authors declare that they have no known competing financial interests or personal relationships which have, or could be perceived to have, influenced the work reported in this article.

## Appendix Supplementary materials

Image, application 1
